# Supplementary material for: High-strength and crack-free welding of 2024 aluminium alloy via Zr-core-Al-shell wire
Source: Nat Commun. 2024 Feb 26;15:1748. doi: 10.1038/s41467-024-45660-x (PMC10897384; doi:10.1038/s41467-024-45660-x)
Supplement: Supplementary file 1 — Supplementary Information [file 41467_2024_45660_MOESM1_ESM.pdf]

Supplementary Information for

High-strength and crack-free welding of 2024 aluminum alloy via Zr-core-Al-shell wire

Jun Jin[1], Shaoning Geng[1,*], Leshi Shu[1], Ping Jiang[1,*], Xinyu Shao[1], Chu Han[1], Liangyuan Ren[1], Yuantai Li[1], Lu Yang[1] & Xiangqi Wang[2]

[1] The State Key Laboratory of Intelligent Manufacturing Equipment and Technology, School of Mechanical Science and Engineering, Huazhong University of Science & Technology, Wuhan, Hubei, 430074, PR China, [2] Jihua Laboratory Testing Center, Ji Hua Laboratory, Foshan, PR China.

*email: sngeng@163.com; jiangping@hust.edu.cn

1. The phase filed (PF) modelling

Supplementary Fig. 1 shows the temperature histories of weld centre at different locations. The average cooling rate was used for phase field (PF) modelling.

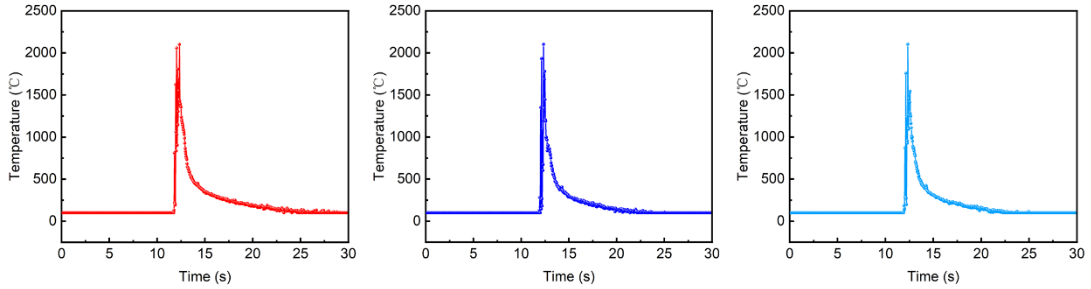

Supplementary Fig. 1. Temperature histories of weld centre with experimental measurement

The phase-field (PF) model developed by Takaki and Ohno[1,2] was adopted in this study. The governing equations of ϕ and u of the PF are as follows:

$$\tau_0 a_s(\vec{n})^2\left[1-(1-k)u'\right]\frac{\partial\phi}{\partial t}=W_0^2\nabla\cdot\left[a_s(\vec{n})^2\nabla\phi\right]+W_0^2\frac{\partial}{\partial x}\left[\left|\nabla\phi\right|^2 a_s(\vec{n})\frac{\partial a_s(\vec{n})}{\partial(\partial_x\phi)}\right]$$
$$+W_0^2\frac{\partial}{\partial y}\left[\left|\nabla\phi\right|^2 a_s(\vec{n})\frac{\partial a_s(\vec{n})}{\partial(\partial_y\phi)}\right]-\frac{df(\phi)}{d\phi}-\lambda\frac{dg(\phi)}{d\phi}(u+u') \tag{1}$$

$$\frac{1}{2}\left[1+k-(1-k)\phi\right]\frac{\partial u}{\partial t}=\nabla\left[D_l q(\phi)\nabla u-j_{AT}\right]+\frac{1}{2}\left[1+(1-k)u\right]\frac{\partial\phi}{\partial t}-\nabla\cdot J \tag{2}$$

Eq. (1) represents the time evolution of the PF. $\tau_0 = a_2\lambda W_0/D_l$ is the relaxation time, W_0 is the interface thickness, and $\lambda = a_1 W_0/d_0$ is the coupling constant associated with the thermodynamic driving force. The numerical constants a_1 and a_2 are $a_1 = 0.8839$ and $a_2 = 0.6267$. $d_0 = k\Gamma/[|m|(1-k)c_0]$ is the chemical capillary length, where k is the partition coefficient, m is the liquidus slope, c_0 is the initial concentration in the liquid, and Γ is the Gibbs–Thomson coefficient. $a_s(\vec{n})$ is the crystalline anisotropy, which is given as:

$$a_s(\vec{n})=(1-3\varepsilon_4)\left[1+\frac{4\varepsilon_4}{1-3\varepsilon_4}\frac{(\partial_x\phi)^2+(\partial_y\phi)^2}{\left|\nabla\phi\right|^4}\right] \tag{3}$$

where ε_4 is the anisotropic strength. $u' = (T - T_s)/(T_l - T_s)$ is the nondimensional temperature undercooling. The polynomials $f(\phi)$ and $g(\phi)$ are given as $df(\phi)/d\phi = -\phi +$

ϕ^3 and $dg(\phi)/d\phi = (1 - \phi^2)^2$. According to the mixture rule, the local concentration c is given by $c = [(1 + \phi)c_s + (1 - \phi)c_l]/2$, where c_l and c_s are the solute concentration in the liquid and solid phases, respectively. c_l and c_s satisfy the relation $k = c_s^e/c_l^e = c_s/c_l$, where c_s^e and c_l^e are the equilibrium concentrations in the solid and liquid, respectively. Eq. (2) represents the time of the solute diffusion equation. Dimensionless supersaturation u is defined as follows:

$$u = \frac{c_l - c_s^e}{(1-k)c_l^e} \tag{4}$$

D_l and D_s are the solute diffusivities in liquids and solids, respectively. j_{AT} is the anti-trapping current, which is given by

$j_{AT} = -(1-kD_s/D_l)(2/\sqrt{2})W_0[1+(1-k)u](\partial\phi/\partial t)\nabla\phi/|\nabla\phi|$. $q(\phi)$ is an interpolating function given by $q(\phi) = [kD_s+D_l+(kD_s-D_l)]\phi/(2D_l)$. **J** is the fluctuating current of a random Gaussian number.

Supplementary Table 1 Physical properties of the Al-4.5 wt% Cu alloy used in the PF simulations[2-4]

Physical properties	Values
Melting temperature of Al (T_m)	933.47 K
Liquidus slope (m)	3.373 K/wt%
Diffusion coefficient in liquid (D_l)	3.0×10^{-9} m^2·s^{-1}
Diffusion coefficient in solid (D_s)	1.0×10^{-12} m^2· s^{-1}
Gibbs-Thompson (Γ)	2.4×10^{-7} K·m
Anisotropy strength of surface energy (ε_4)	0.02
Mean nucleation undercooling (ΔT_N)	3.0 K

2. Estimating the strengthening contributions

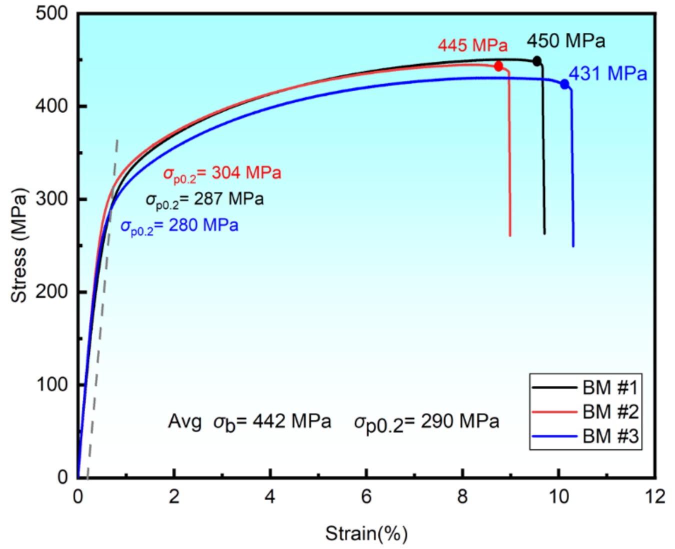

Supplementary Fig. 2. Engineering stress-strain curves of the base metals. Tests for three samples are shown (BM #1, BM #2 and BM #3) along with their 0.2% yield point (dashed line).

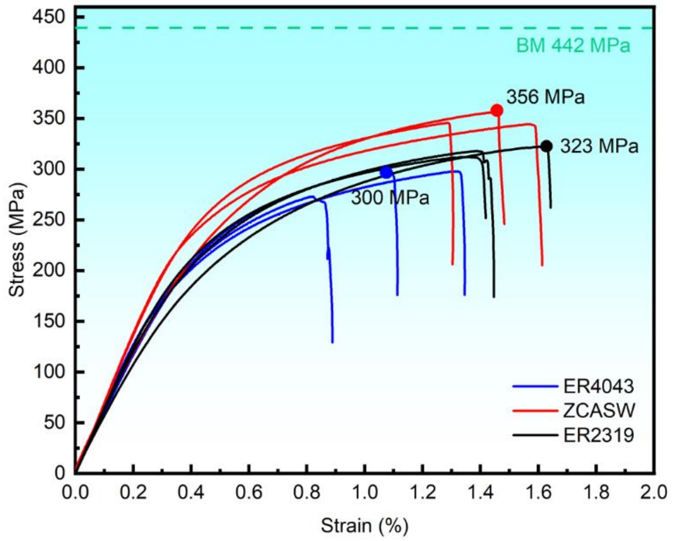

Supplementary Fig. 3. Engineering stress-strain curves of the welded joints fabricated with different filler materials. Tests for three samples under each filler material (ER4043, ER2319 and ZCASW) are shown along with the average tensile strength of base metal.

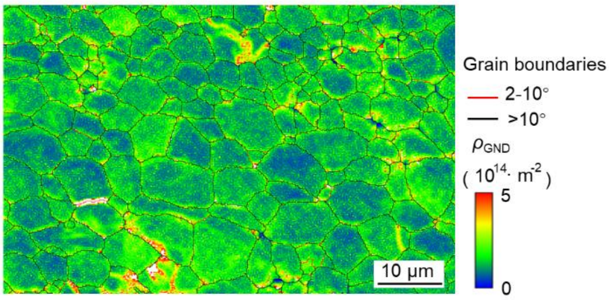

Supplementary Fig. 4. Distribution of the GND density of the melting zone fabricated with the ZCASW filler.

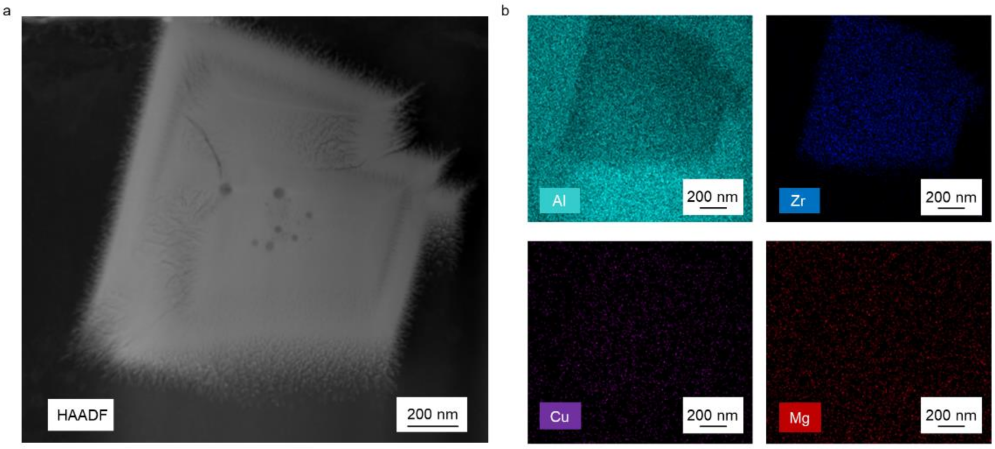

Supplementary Fig. 5. TEM-EDX characteristics of precipitates in the dendrite interior of the melting zone fabricated with the ZCASW filler. a STEM-HAADF mode. **b** Corresponding EDX mapping image.

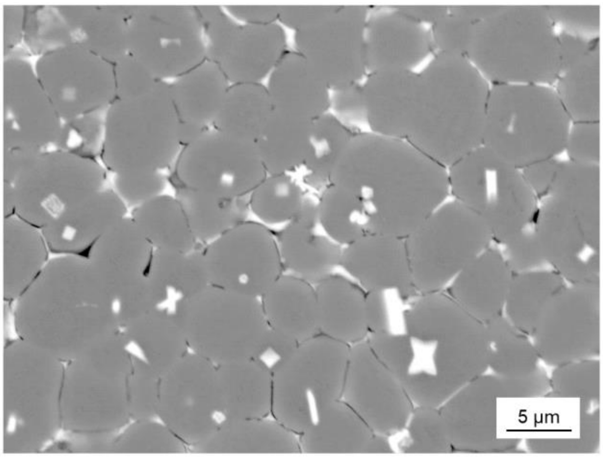

Supplementary Fig. 6. SEM (BSE mode) characteristics of the melting zone fabricated with the ZCASW filler.

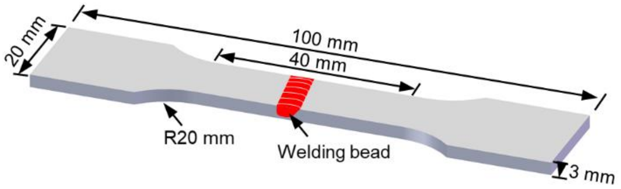

Supplementary Fig. 7. The schematic diagram of tensile specimen.

Supplementary Table 2 SEM-EDS measurements of primary dendrites of the melting zone fabricated with ZCASW filler/wt%

Elements	Point 1	Point 2	Point 3	Point 4	Point 5	Average
Al	95.6	95.6	94.5	95.5	93.4	94.92
Mg	2.0	2.1	2.3	1.9	2.3	2.12
Cu	1.4	1.5	2.3	1.5	3.4	2.02
Ti	0.2	0.2	0.2	0.2	0.2	0.2
Mn	0.7	0.7	0.7	0.8	0.7	0.72

Supplementary Table 3 Chemical composition of deposit fabricated with the ZCASW filler/wt%

Composition	Al	Cu	Mg	Ti	Mn	Zr
Deposit	91.7117	4.1957	1.4899	0.0653	0.4934	1.8066

Supplementary References

1. Takaki, T. et al. Competitive grain growth during directional solidification of a polycrystalline binary alloy: Three-dimensional large-scale phase-field study. *Materialia*. **1**, 104-113 (2018).
2. Takaki, T. et al. Two-dimensional phase-field simulations of dendrite competitive growth during the directional solidification of a binary alloy bicrystal. *Acta Mater.* **81**, 272-283 (2014).
3. Ahmadein, M. et al. Grain Nucleation Parameters for Aluminum Alloys: Experimental Determination and Model Validation. *Metall Mater Trans. A.* **40**, 646-653 (2009).
4. Han, C. et al. Ultra grain refinement and mechanical properties improvement of all-weld-metal for medium-thick Al-Li alloy via laser beam oscillation and in-situ alloying. *Opt. Laser Technol.* **168**, 109965 (2024).
